# The Efficacy and Safety of Multi-Kinase Inhibitors in Adrenocortical Carcinoma: A Systematic Review and Single-Arm Meta-Analysis

**DOI:** 10.3390/cancers17122004

**Published:** 2025-06-16

**Authors:** Fabiano Flauto, Vincenzo Damiano

**Affiliations:** Department of Clinical Medicine and Surgery, University of Naples Federico II, 80131 Naples, Italy; fabiano.flauto@unina.it

**Keywords:** adrenocortical carcinoma, target therapy, immuno-oncology, meta-analysis

## Abstract

**Background:** Advanced adrenocortical carcinoma (ACC) remains a challenging malignancy with limited therapeutic options. Multi-kinase inhibitors (MKIs), either alone or in combination with immuno-oncology (IO) agents, have been investigated in recent single-arm clinical trials and retrospective series. **Methods:** We conducted a systematic review and single-arm meta-analysis of studies evaluating MKIs in advanced ACC. Objective response rate (ORR) and disease control rate (DCR) were pooled using random-effects models for single-arm proportions. Overall survival (OS) and progression-free survival (PFS) were summarized descriptively due to limited variance data. Subgroup analyses compared MKI monotherapy versus MKI + IO combinations, and meta-regression was performed to assess the impact of prior mitotane exposure. **Results:** Eleven studies (*n* = 208 patients) were included. The pooled ORR was 21% (95%CI, 11–36%), and the DCR was approximately 57%. Subgroup analysis revealed a higher ORR with MKI + IO regimens (26%; 95%CI, 12–48%) compared to MKI monotherapy (15%; 95%CI, 3–47%). Median OS ranged from 5.4 to 30.6 months, and PFS from 2.8 to 13.3 months, both favouring MKI + IO combinations. Meta-regression identified prior mitotane exposure as a significant predictor of ORR (*p* = 0.0279), particularly within the MKI + IO subgroup. **Conclusions:** MKI-based regimens, especially when combined with IO, demonstrate promising efficacy in advanced ACC, a disease with few established second-line options. While limited by the non-comparative design of available studies, these findings support further investigation in prospective, randomized clinical trials.

## Simple Summary

Adrenocortical carcinoma (ACC) is a rare and aggressive malignancy with limited therapeutic options and poor prognosis in advanced stages. Recent interest has focused on multi-kinase inhibitors (MKIs), particularly in combination with immuno-oncology (IO) agents, as a strategy to enhance antitumor efficacy. We performed a systematic review and single-arm meta-analysis to evaluate the clinical activity of MKI-based regimens in advanced ACC. Our results indicate that MKIs, especially when combined with IO, are associated with improved objective response rates and prolonged survival compared to historical outcomes. Notably, prior exposure to mitotane emerged as a potential predictor of enhanced response, particularly in patients receiving combination therapy. These findings underscore the potential of MKI-based strategies in ACC and support further prospective investigation to optimize patient selection and treatment sequencing.

## 1. Introduction

Adrenocortical carcinoma (ACC) is a rare and aggressive endocrine malignancy associated with poor prognosis and limited treatment options, particularly in locally advanced or metastatic stages. The current standard of care includes mitotane, either alone or combined with cytotoxic chemotherapy, with modest efficacy and generally unsatisfactory survival outcomes [1,2]. Despite decades of investigation, progress in systemic therapy for ACC has been limited, primarily due to the disease’s rarity, biological heterogeneity, and lack of randomized clinical trials. Recent advances in precision oncology have renewed interest in targeted therapies for ACC, particularly multi-kinase inhibitors (MKIs), which inhibit angiogenic and proliferative signaling pathways such as VEGFR, PDGFR, and RET [3]. Agents like cabozantinib, lenvatinib, sunitinib, and anlotinib have shown preliminary activity in small Phase II trials and retrospective series [4,5,6,7,8,9,10,11]. However, the efficacy of MKIs as a monotherapy remains inconsistent, with low objective response rates (ORRs) and limited disease stabilization. To improve outcomes, combination strategies have emerged, notably the integration of immune-oncology (IO) with MKIs, leading to a growing number of early-phase studies evaluating MKI + IO regimens, though current data remain fragmented and largely non-randomized [11,12,13,14]. In this context, a comprehensive synthesis is needed to clarify the efficacy and safety of MKI-based therapies, both alone and in combination with IO. Identifying predictors of response, such as prior mitotane exposure, may further guide patient selection. This systematic review and single-arm meta-analysis aim to assess ORR, disease control rate (DCR), and survival outcomes, including overall survival (OS) and progression-free survival (PFS), of MKI-based therapies in advanced ACC.

## 2. Materials and Methods

### 2.1. Registration and Search Strategy

This systematic review and meta-analysis was performed in accordance with the Preferred Reporting Items for Systematic Reviews (PRISMA) guidelines for meta-analysis, whose checklist is provided in Supplementary Table S1 [15].

Two independent reviewers (FF, VD) screened titles and abstracts for relevance. The software “Rayyan” [16] was used to assess the suitability of screened papers for inclusion. The final studies selection was documented following the PRISMA guidelines flow diagram methodology [15]. The protocol was registered in the International Prospective Register of Systematic Reviews (PROSPERO) with the following registration number: CRD420251017632. PubMed, Scopus, Central, ASCO, ESMO, ENSAT, Embase, Web of Science, and Clinicaltrial.gov were the selected databases to be systematically screened. Updates of included studies were manually searched up to 30 March 2025 and included in the analysis. The search string is shown in Supplementary Table S2. The ORR and DCR rates were calculated using proportional meta-analysis. The degree of statistical heterogeneity between studies was evaluated using the I^2^ statistic. A fixed-effects model was employed if there was no statistical heterogeneity (I^2^ < 50%) across the studies. A random-effects model was utilized if there was statistical heterogeneity (I^2^ ≥ 50%) between studies.

### 2.2. Inclusion and Exclusion Criteria

Trials evaluating the efficacy of MKI monotherapy or MKI + IO in patients with ACC were considered eligible. Trials investigating the addition of loco-regional treatments to systemic regimens were excluded. Eligible studies were required to report an efficacy analysis for patients with ACC. For studies with data published in multiple reports, the most recent update, as of the data cut-off on 30 March 2025, was selected.

### 2.3. Data Extraction and Definitions

From each study, we extracted data on study design (retrospective, prospective, or Phase II), treatment regimen (MKI or MKI + IO), sample size, percentage of patients with prior mitotane exposure, and outcomes of interest (ORR, DCR, OS, PFS). Studies were categorized into two subgroups based on treatment strategy (MKI and MKI + IO). Included study characteristics are provided in Table 1.

### 2.4. Risk of Bias Assessment

Two reviewers independently assessed the risk of bias in each study. Discrepancies between the reviewers’ judgments throughout the process were resolved according to discussions among reviewers. The methodological index for non-randomized studies (MINORS) tool was used for case series, non-randomized controlled trials, and single-arm clinical trials [17]. The MINORS evaluation criteria items that we used to assess the quality of the studies included (1) a clear aim, (2) the inclusion of patients in a consecutive manner, (3) prospectively collected clinical data, (4) appropriate endpoints, (5) an unbiased assessment of endpoints, (6) an appropriate follow-up time, (7) less than 5% loss during follow-up, and (8) a calculation of the study size before study initiation. In the case of single-arm studies, the following items were not applicable: (1) a suitable control arm, (2) contemporary groups, (3) baseline similarity between arms, and (4) appropriate statistical analyses. The items were scored 0 if not reported, 1 if reported but inadequate, or 2 if reported and adequate. We considered scores of 12 points or more high quality with a low risk of bias; scores between 8 and 12 points at medium risk of bias; and scores of 8 points or less at high risk of bias. A graphical summary of the risk of bias assessment is shown in Supplementary Table S3. We also assessed publication bias via a visual inspection of funnel plots and conducted Egger’s test and Begg’s test using the ‘meta’ package in R (v4.4.2) (Supplementary Figure S1).

### 2.5. Statistical Analysis

All statistical analyses were performed using “R version 4.4.2” with relevant packages, including “meta”, “metafor”, “dmetar”, and “ggplot2” [18]. Given the absence of randomized or matched comparative data across all included studies, a single-arm meta-analytic framework was chosen based on its suitability to synthesize evidence from non-comparative studies. This methodological approach focuses on the aggregation of absolute treatment effects (ORR, DCR, PFS, OS) from individual arms, without requiring a control group within each study. By pooling these absolute estimates across studies, the method provides a robust estimate of average treatment efficacy. While it does not allow for direct causal inference, it offers a valid statistical foundation for exploratory subgroup comparisons and indirect hypothesis generation.

Proportional outcomes, such as ORR and DCR, were pooled using the metaprop function from the meta package. Proportions were logit-transformed to stabilize variances, and pooled estimates were calculated using a random-effects model (DerSimonian-Laird method). Heterogeneity was quantified using the I^2^ statistic, τ^2^ (tau-squared), and Cochran’s Q test. For time-to-event outcomes (OS and PFS), the “metamean” function was used to compute pooled means where appropriate. However, due to a lack of consistent reporting of measures of dispersion (e.g., standard deviation or confidence intervals of medians), a narrative and descriptive summary was provided for median OS and PFS across studies. Supplementary visual summaries (bar plots) were constructed to highlight differences in survival outcomes by treatment group (MKI vs. MKI + IO).

Subgroup analyses were conducted to compare outcomes between MKI monotherapy and MKI + IO regimens. Between-group differences were assessed using both fixed-effect and random-effects models where applicable.

#### 2.5.1. Descriptive Analysis

Basic summary statistics were calculated for study characteristics and efficacy endpoints. Median and mean values were reported for OS and PFS. The visualization of efficacy metrics across studies (ORR, OS, PFS) was performed using bar plots and grouped plots.

#### 2.5.2. Meta-Analysis of Proportions (ORR and DCR)

Single-arm meta-analyses were conducted for ORR and DCR using the metaprop() function. The number of responders was calculated by multiplying reported ORR or DCR percentages by the total number of patients in each study. Proportions were pooled using random-effects models, with inverse variance weighting. The Freeman-Tukey double arcsine transformation (PLOGIT) was applied to stabilize variances.

#### 2.5.3. Subgroup Analysis by Treatment Strategy

Subgroup analyses were conducted to compare efficacy outcomes between MKI-only and MKI + IO regimens using the byvar argument in meta-analysis functions. Separate pooled estimates were generated for each group. Between-group differences were assessed using fixed- and random-effects models.

#### 2.5.4. Descriptive Analysis of Time-to-Event Outcomes (OS and PFS)

Due to lack of reported standard errors and the single-arm design of included studies, formal survival meta-analysis was not feasible. Median OS and PFS values were extracted and summarized descriptively. Group-level averages and ranges were compared across MKI and MKI + IO studies.

#### 2.5.5. Supplementary Survival Analysis

Kaplan-Meier (KM)-derived survival probabilities at 6 and 12 months were extracted from published curves when available. Pooled estimates of time-specific survival were calculated using single-arm meta-analysis, and subgroup analyses were performed by treatment group (MKI vs. MKI + IO).

#### 2.5.6. Sensitivity Analysis

To assess the robustness of pooled ORR, a sensitivity analysis was conducted by excluding retrospective studies and including only prospective and Phase II studies. The pooled ORR estimate was recalculated in this subset using the same random-effects model.

#### 2.5.7. Meta-Regression on Prior Mitotane Exposure

Meta-regression analyses were performed using the rma function in the metafor package to explore potential sources of heterogeneity in ORR. Covariates included prior mitotane exposure (as a percentage), median age, study design (retrospective vs. prospective), and treatment type (MKI vs. MKI + IO). A multivariable mixed-effects meta-regression model was fitted, and the proportion of between-study variance explained (R^2^) was reported. Bubble plots were used to visualize the relationship between prior mitotane exposure and ORR, with bubble size representing study weight (inverse standard error). Separate meta-regression analyses were also conducted within treatment subgroups (MKI and MKI + IO) to assess effect modification by prior mitotane exposure. The metareg() function was used to regress logit-transformed ORR on prior mitotane percentage. The regression coefficient, confidence interval, *p*-value, and R^2^ statistic (proportion of between-study heterogeneity explained) were reported.

## 3. Results

### 3.1. Study Selection

**Figure 1:**
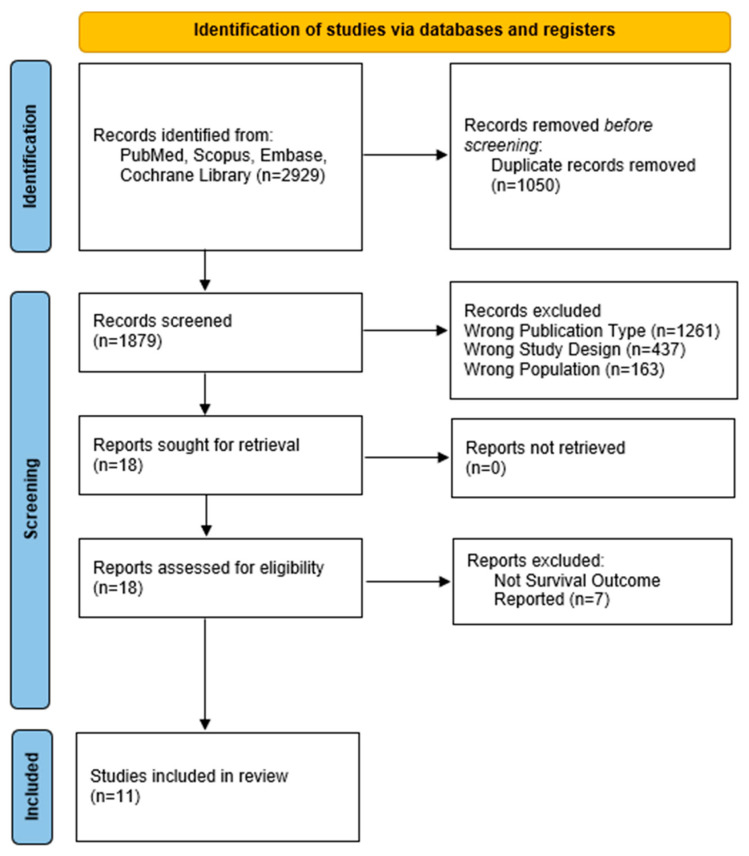
PRISMA flow diagram.

A total of 11 studies (patients = 208) evaluating MKIs, either as monotherapy or in combination with IO, were included in this analysis. These studies were conducted in patients with locally advanced or metastatic ACC and consisted of Phase II single-arm trials or retrospective cohorts, reflecting the rarity of the disease and challenges in conducting large, randomized trials. The median sample size across studies was 17 patients (range: 7–39). PRISMA flow diagram summarizing the study selection is provided in Figure 1.

### 3.2. Efficacy Analysis and Forrest Plot

**Figure 2:**
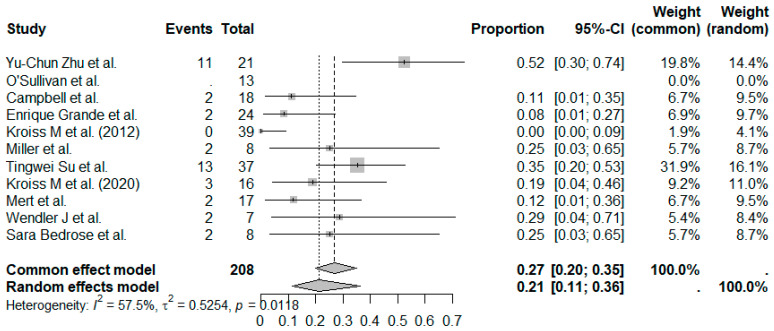
Proportion analysis for ORR in all 11 studies. The random-effect models’ pooled ORR was 21%. References: Yu-Chun Zu et al. [14], O’Sullivan et al. [11], Campbell et al. [6], Enrique Grande et al. [13], Kroiss M et al. (2012) [4], Miller et al. [12], Tingwei Su et al. [7], Kroiss M et al. (2020) [5], Mert et al. [9], Wendler J et al. [8], Sara Bedrose et al. [10].

**Figure 3:**
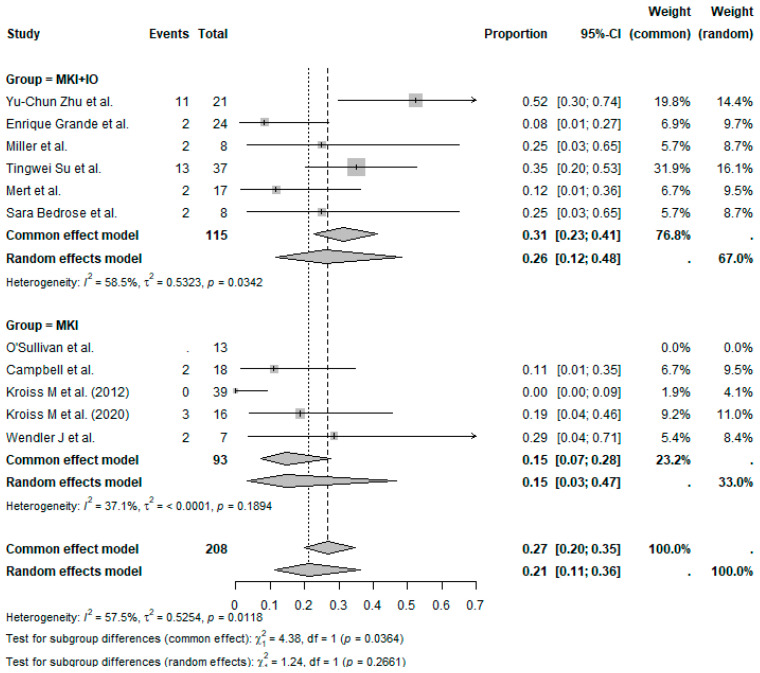
Proportion analysis ORR according to treatment group (MKI + IO and MKI). References: Yu-Chun Zu et al. [14], O’Sullivan et al. [11], Campbell et al. [6], Enrique Grande et al. [13], Kroiss M et al. (2012) [4], Miller et al. [12], Tingwei Su et al. [7], Kroiss M et al. (2020) [5], Mert et al. [9], Wendler J et al. [8], Sara Bedrose et al. [10].

The pooled ORR across all studies was 21% (95%CI, 11–36%), with moderate heterogeneity (I^2^ = 57.5%) (Figure 2). Subgroup analysis based on treatment regimen showed a pooled ORR = 26% (95%CI, 12–48%) in MKI + IO group (*n* = 6 studies) and a pooled ORR = 15% (95%CI, 3–47%) in MKI group (*n* = 5 studies) (Figure 3). Although the pooled ORR appeared numerically higher in the combination group, the test for subgroup differences was not statistically significant under the random-effects model (*p* = 0.2661). However, under the common-effect model, the difference did reach significance (*p* = 0.0364), suggesting a possible benefit with the addition of immunotherapy that merits further exploration.

A sensitivity analysis was conducted including only prospective or Phase II trials (*n* = 5 studies) to evaluate the robustness of the pooled ORR. The pooled ORR in this subset was 16% (95%CI, 5–41%; I^2^ = 77.2%, *p* = 0.0015), compared to 21% in the overall analysis (Supplementary Figure S2). This suggests that inclusion of retrospective studies may modestly inflate pooled efficacy estimates. However, considerable heterogeneity persisted despite study type restriction.

**Figure 4:**
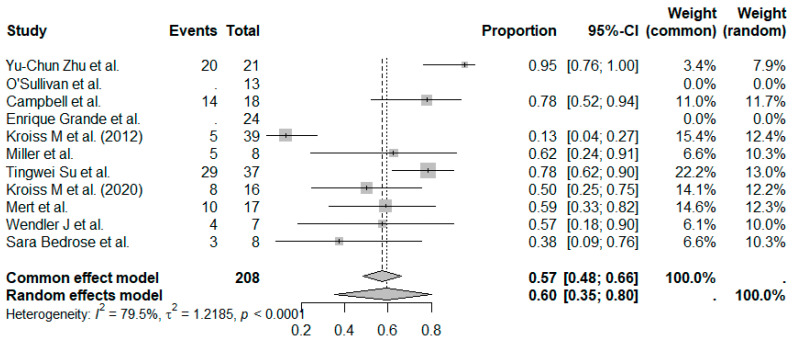
Proportion analysis for DCR in all 11 studies. The random-effect models’ pooled DCR was 60%. References: Yu-Chun Zu et al. [14], O’Sullivan et al. [11], Campbell et al. [6], Enrique Grande et al. [13], Kroiss M et al. (2012) [4], Miller et al. [12], Tingwei Su et al. [7], Kroiss M et al. (2020) [5], Mert et al. [9], Wendler J et al. [8], Sara Bedrose et al. [10].

**Figure 5:**
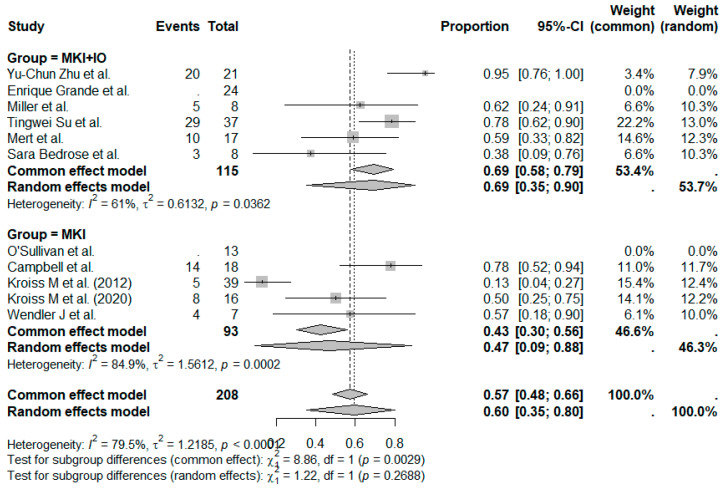
Proportion analysis DCR according to treatment group (MKI + IO and MKI). References: Yu-Chun Zu et al. [14], O’Sullivan et al. [11], Campbell et al. [6], Enrique Grande et al. [13], Kroiss M et al. (2012) [4], Miller et al. [12], Tingwei Su et al. [7], Kroiss M et al. (2020) [5], Mert et al. [9], Wendler J et al. [8], Sara Bedrose et al. [10].

The pooled DCR across all studies was estimated at 60% (95%CI, 39–73%), with moderate between-study heterogeneity (Figure 4). When stratified by treatment type, the MKI + IO group demonstrated a pooled DCR of 69%, while the MKI group showed a pooled DCR of 47% (Figure 5). Although formal subgroup analysis was not feasible due to limitations in reporting across studies, the trend favored combination therapy, consistent with the observed ORR outcomes.

Due to the lack of individual-level data and consistent variance reporting, a formal pooled meta-analysis of OS was not feasible. Therefore, a descriptive analysis was performed using reported median OS (Supplementary Figure S3).

A total of 10 studies were included in the descriptive analysis of survival outcomes, comparing MIKs alone to MKI + IO combinations. Among studies evaluating MKIs alone (*n* = 5), the median OS ranged from 5.4 to 26.9 months, with a mean OS of 15.8 months. In contrast, MKI + IO studies (*n* = 5) reported a longer OS range of 13.5 to 30.6 months (mean median OS = 20.9 months).

PFS was also summarized descriptively due to the same limitations noted for OS. In the MKI monotherapy group (*n* = 5 studies), median PFS ranged from 2.8 to 6.0 months, with a mean of medians of 4.6 months. In the MKI + IO group (*n* = 6 studies), PFS was more favorable, ranging from 2.9 to 13.3 months, with a mean of medians of 6.7 months. As with OS, the numerical advantage observed in the MKI + IO group supports a possible additive or synergistic effect of combining targeted therapy with immune modulation, though interpretation should be cautious due to the descriptive nature of the analysis.

**Figure 6:**
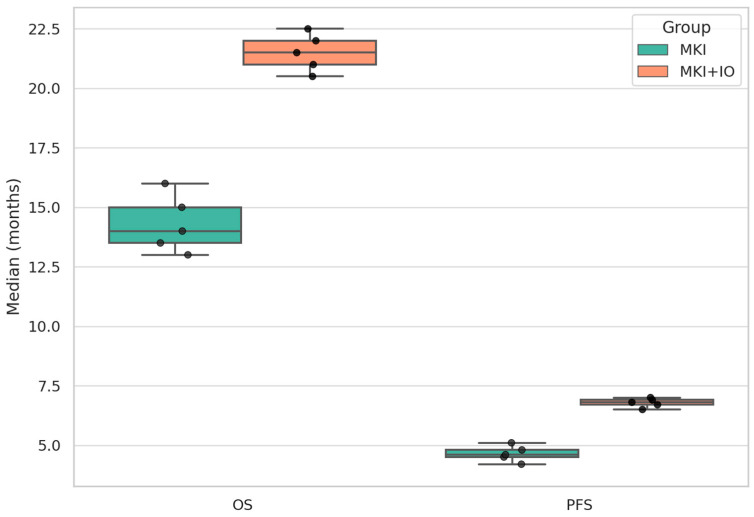
Descriptive distribution of median OS and PFS by treatment group (MKI vs. MKI + IO). Each black dot represents the median survival outcome reported in an individual study. Boxplots illustrate the interquartile range, with horizontal bars indicating medians. Whiskers extend to 1.5×IQR. No inferential comparisons were performed, and some medians are reported without associated confidence intervals. This figure provides a visual summary of the heterogeneity across studies and the trend toward longer OS and PFS in the MKI + IO group.

To enhance interpretability, a comparative bar plot (Figure 6) illustrates the median OS and PFS values by treatment group. The graphic highlights the consistent numerical advantage of the MKI + IO combinations across both survival endpoints. While the data are descriptive and not derived from direct comparisons within randomized controlled trials, the visual representation reinforces the trend toward improved efficacy with combined treatment strategies.

**Figure 7:**
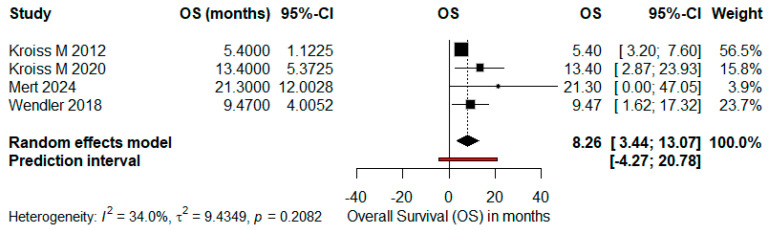
Forrest plot for OS, indicating a pooled estimate of 8.26 months (95%CI, 3.44–13.07 months), highlighting moderate heterogeneity (I^2^ = 34%; *p* = 0.2082). References: Kroiss M et al. (2012) [4], Kroiss M et al. (2020) [5], Mert et al. [9], Wendler J et al. [8].

**Figure 8:**
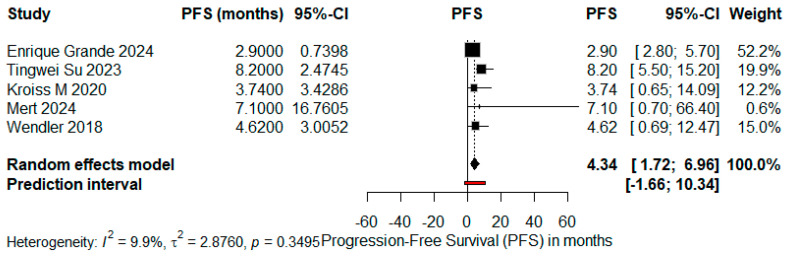
Forrest plot for PFS, reporting a pooled estimate of 4.34 months (95%CI, 1.72–6.96 months), with minimal heterogeneity (I^2^ = 9.9%; *p* = 0.3495). References: Enrique Grande et al. [13], Tingwei Su et al. [7], Kroiss M et al. (2020) [5], Mert et al. [9], Wendler J et al. [8].

Forrest plots for PFS and OS were constructed considering only trials with complete data, facilitating the precise estimation of effect size and associated confidence intervals. The meta-analysis applied a random-effects model, taking into account inter-study variability, as represented by the prediction intervals and heterogeneity statistics (I^2^, τ^2^, and *p*-values) (Figure 7 and Figure 8).

### 3.3. Meta-Regression

**Figure 9:**
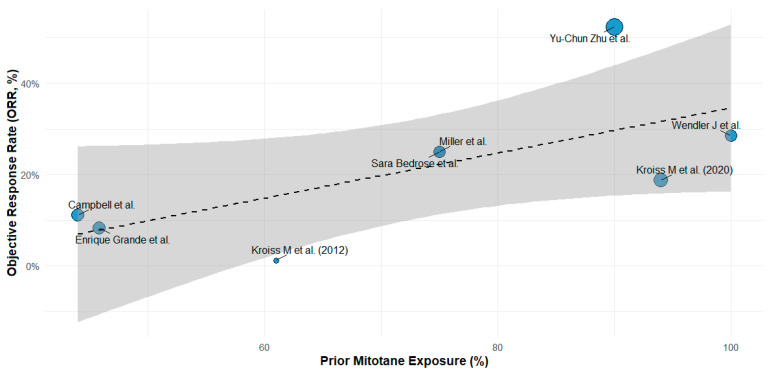
Meta-regression bubble plot illustrating the relationship between prior mitotane exposure and ORR across included studies. Each bubble represents a study, with size proportional to the inverse of the standard error. A significant positive association was observed (*p* = 0.0279), suggesting prior mitotane exposure as a potential predictor of therapeutic response. References: Yu-Chun Zu et al. [14], Campbell et al. [6], Enrique Grande et al. [13], Kroiss M et al. (2012) [4], Miller et al. [12], Kroiss M et al. (2020) [5], Wendler J et al. [8], Sara Bedrose et al. [10].

A univariate meta-regression was performed to evaluate the relationship between prior mitotane exposure and observed ORR across the included studies (Figure 9). Eight studies with available data were analyzed. Prior mitotane exposure significantly correlated with increased ORR (slope = 0.0349; 95%CI, 0.0038–0.0661; *p* = 0.0279), explaining approximately 60% of between-study heterogeneity (R^2^ = 59.8%). Moderate residual heterogeneity remained (I^2^ = 33%). These results suggest that higher prior mitotane exposure may predict improved response rates to subsequent MKI-based therapies, potentially reflecting treatment sequencing effects or underlying biological selection.

**Figure 10:**
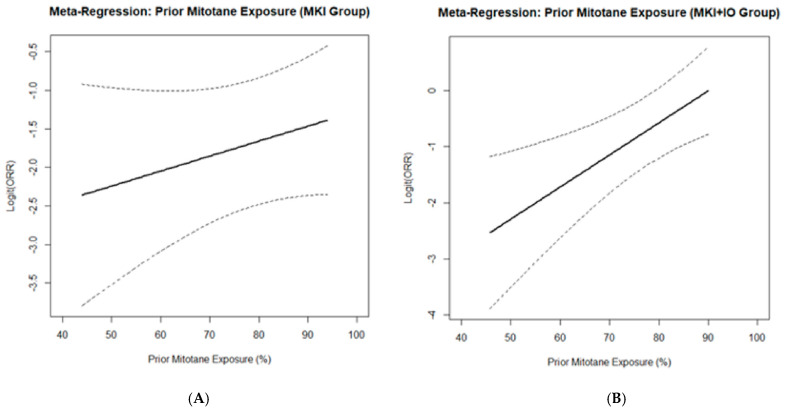
Subgroup meta-regression analyses assessing the association between prior mitotane exposure and ORR separately for MKI + IO studies (**Panel A**) and MKI monotherapy studies (**Panel B**). The *x*-axis indicates the percentage of patients with prior mitotane exposure, while the *y*-axis shows logit-transformed ORR. The solid black lines represent regression trends, with dashed lines indicating 95%CI. A significant positive correlation was found only in the MKI + IO subgroup (*p* = 0.0031).

Subsequently, subgroup analyses were performed to explore this relationship separately in studies involving MKI and MKI + IO combination therapy (Figure 10). A significant positive association between prior mitotane exposure and ORR was observed in the MKI + IO group (*p* = 0.0031), while no significant correlation emerged within the MKI monotherapy subgroup (*p* = 0.2592). These findings suggest that prior mitotane exposure may specifically enhance responsiveness to subsequent immunotherapy-containing regimens.

### 3.4. Supplementary Survival Analysis

For a subset of 8 studies reporting KM curves, survival probabilities at fixed time points (6 and 12 months) for both PFS and OS were extracted. A single-arm meta-analysis of survival rates at 6 and 12 months was conducted using the metaprop() function. Forrest plots were generated to illustrate pooled survival probabilities at each time point (Supplementary Figures S4–S7). Additional subgroup analyses were performed to compare MKI-only versus MKI + IO regimens. This approach allowed us to provide a clinically interpretable summary of survival likelihood over time, complementing the median survival results. These findings further support trends in improved outcomes among patients treated with MKI + IO combinations.

## 4. Discussion

This systematic review and single-arm meta-analysis represents one of the most comprehensive evaluations of MKIs in advanced ACC to date. Standard therapies, primarily mitotane combined with cytotoxic chemotherapy, offer limited clinical benefits, with ORRs around 23% and median PFS near 5 months [19]. Our findings indicate that MKIs, modestly effective alone, achieve significantly improved outcomes when combined with IO. Specifically, MKI + IO combinations demonstrated numerically higher pooled ORRs and trends toward enhanced OS and PFS compared to MKI monotherapy. Notably, VEGFR-targeting MKIs plus PD-1 inhibitors, such as apatinib combined with camrelizumab, reported ORRs exceeding 50% and DCRs greater than 90%, surpassing historical benchmarks [14].

This clinical synergy between MKIs and IO is biologically plausible. MKIs targeting VEGFR pathways can normalize tumor vasculature, reduce hypoxia, and facilitate immune cell infiltration by mitigating immunosuppressive signaling in the tumor microenvironment. These changes are particularly relevant in ACC, generally considered an immunologically “cold” tumor, potentially sensitizing it to IO. Preclinical studies support this mechanism, demonstrating MKIs’ capacity to downregulate PD-L1, suppress regulatory T-cell function, and exert direct cytostatic effects, collectively enhancing antitumor immunity [20,21,22].

An intriguing finding from our meta-regression analysis was that prior mitotane exposure positively correlated with higher ORR, particularly in the MKI + IO subgroup. This observation is counterintuitive given pharmacological evidence indicating that mitotane-induced cytochrome P450 3A4 activity accelerates MKI metabolism, theoretically reducing efficacy [23,24,25,26]. Several hypotheses might explain this paradox: first, ecological bias due to aggregated study-level data may have introduced confounding, as studies with higher mitotane exposure might involve patients with less aggressive disease or superior clinical management; second, treatment sequencing may have mitigated drug-drug interactions, with mitotane potentially discontinued before MKI + IO initiation; third, prior mitotane exposure could serve as a proxy for favorable prognosis or treatment tolerance, or might possess immunomodulatory properties, enhancing responsiveness to MKI + IO.

Nonetheless, this observation remains hypothesis-generating. Considerable variability in how mitotane exposure was defined (dose, timing, duration) limited cross-study consistency. The use of aggregated study-level data further precludes adjustment for key confounders such as sequencing, pharmacokinetics, or clinical status. These factors likely introduced bias. While meta-regression explored mitotane as a potential moderator, lack of standardized definitions and individual-level data restricts interpretation.

Our analysis has several limitations. Given the absence of randomized or matched control arms, this study employed a single-arm meta-analytic framework to synthesize absolute treatment effects from non-comparative studies. Pooled estimates of ORR, DCR, OS, and PFS reflect aggregate outcomes across heterogeneous ACC cohorts, offering a pragmatic synthesis tool in ultra-rare cancers where randomized trials are often unfeasible. Subgroup analyses comparing MKI monotherapy and MKI + IO were performed to explore potential efficacy differences. However, these comparisons are exploratory and should be interpreted with caution due to the lack of individual patient-level data (IPD) and unaccounted confounders such as baseline characteristics or treatment sequencing.

The differences in efficacy between MKI monotherapy and MKI + IO combinations may reflect not only treatment effect but also variations in patient selection or baseline characteristics. Patients receiving combination therapy may have had better performance status, lower disease burden, or more favorable tumor immunobiology. Without IPD, adjustment for these confounders was not possible, limiting causal inference. These limitations underscore the need for cautious interpretation of subgroup analyses and highlight the importance of future prospective trials that account for baseline heterogeneity in study design.

Regarding safety profiles, MKI monotherapy consistently demonstrated high-grade (≥3) adverse events in approximately 60–80% of patients, notably hepatic toxicity, hypertension, hypophosphatemia, diarrhea, mucositis, and fatigue. A clinically relevant interaction was noted between mitotane and MKIs, potentially reducing MKI efficacy and toxicity via CYP3A4 induction [27]. In contrast, MKI + IO combinations showed similar or slightly improved tolerability. For instance, in CABATEN trial, cabozantinib plus atezolizumab had a relatively low incidence (30%) of grade ≥ 3 events, primarily hypertension and rash [13]. Similarly, camrelizumab plus apatinib reported grade 3–4 events in 43% of patients, primarily hepatotoxicity and lymphopenia. Treatment-related fatal events were absent across studies, with adverse events effectively managed through dose adjustments or treatment interruptions [14].

Despite heterogeneity in adverse event reporting, available evidence, particularly from prospective studies, suggests that MKI + IO regimens are generally well-tolerated. These findings support the clinical feasibility of combination therapies, though confirmation in larger, prospectively monitored cohorts with standardized toxicity assessment remain essential.

Predictive biomarkers in ACC remain limited. PD-L1 expression, microsatellite instability (MSI), and tumor mutational burden (TMB) have not reliably predicted responses in ACC, although MSI-high cases have demonstrated sensitivity to IO. Low tumor-infiltrating lymphocytes (TILs) correlate with poor response, while immune-rich molecular subtypes and higher T-cell infiltration may predict benefit [20,21,22]. Conversely, Wnt/β-catenin activation and TP53 mutations contribute to immune exclusion and resistance. Mitotane-induced CYP3A4 activation may further affect MKI pharmacokinetics and efficacy [25,26]. Integrated biomarker models combining genomic and immune characteristics are likely needed to accurately predict treatment response [22,23,24].

Multiple intrinsic and extrinsic resistance mechanisms limit MKI and immunotherapy efficacy in ACC. Immunoevasive tumor microenvironments characterized by aberrant Wnt/β-catenin signaling or TP53 inactivation prevent adequate immune cell infiltration [28,29]. Strategies to overcome resistance include combination immunotherapies (e.g., adding CTLA-4 or LAG-3 inhibitors) and immune-modulating agents targeting specific pathways or enhancing immune activation [19,20,21,22,23,24,29].

Despite their potential, PD-L1, MSI, and TMB could not be included in subgroup analyses due to inconsistent and incomplete reporting. Only one of eleven trials systematically assessed PD-L1 and TMB in relation to clinical outcomes [14]. Most studies either omitted these biomarkers or lacked stratified efficacy data, and differences in assay platforms and thresholds further limited comparability.

Prospective randomized trials stratified by molecular and immune biomarkers, alongside pharmacokinetic assessments and harmonized reporting standards, are critical to validating efficacy and refining precision oncology approaches in this challenging malignancy.

## 5. Conclusions

In conclusion, this systematic review and single-arm meta-analysis provides a comprehensive synthesis of MKI efficacy in advanced ACC. Our findings confirm that MKI monotherapy offers modest but clinically relevant activity, with pooled ORR around 15% and median PFS typically below 6 months, reaffirming its role as a therapeutic option where other systemic strategies fail. The combination of MKIs with IO demonstrated a clear numerical advantage across all efficacy endpoints, including higher ORR (up to 26%), improved disease control, and extended OS and PFS, suggesting synergistic potential between antiangiogenic and immunomodulatory pathways. Although derived from non-comparative studies, these results support the growing rationale for MKI + IO regimens as a promising strategy in ACC. Notably, our meta-regression identified prior mitotane exposure as a significant positive predictor of response, particularly in MKI + IO-treated patients, raising intriguing questions about potential biological priming or treatment sequencing effects. While these observations require cautious interpretation and prospective validation, they highlight the need to consider prior therapeutic exposures when designing future trials and in clinical decision-making.

Overall, despite the inherent limitations of current evidence, our analysis reinforces the evolving role of MKI-based therapies, both as monotherapy and in combination with immunotherapy, in a disease with historically limited options. Rigorous prospective trials, ideally biomarker-driven, are now essential to confirm these signals and to refine patient selection strategies, ultimately advancing toward precision oncology for ACC.

## Figures and Tables

**Table 1:** Summary of included studies evaluating MKI-based treatments in advanced adrenocortical carcinoma. Study characteristics and reported outcomes are listed, including prior mitotane exposure, treatment strategy, and endpoints such as ORR, DCR, PFS, and OS.

Study	Design	Drugs	Population	Prior Mitotane (%)	Primary End Point	ORR (%)	DCR (%)	PFS (Months)	OS (Months)	Follow-Up Time (Months)
Yu-Chun Zhu 2024 [14]	Phase II open label	Apatinib + Camrelizumab	21	90%	ORR	52 (95%CI, 30–74%)	95 (95%CI, 76–100)	13.3 (95%CI, 8.4-NR)	20.9 (95%CI, 11.0-NR)	17.5
O’Sullivan 2014 [11]	Phase II open label	Axitinib	13	NA	NA	NA	NA	5.48 (3.67-NR)	26.92 (9.05-NR)	NA
Campbell 2024 [6]	Phase II open label	Cabozantinib	18	44	PFS4	11	78	6 (95%CI, 4.3-NR)	24.0 (95%CI, 15.6-NR)	36.8
Enrique Grande 2024 [13]	Phase II open label	Cabozantinib + Atezolizumab	24	45.8	ORR	8.3 (95%CI, 1–27)	NA	2.9 (95%CI, 2.8–5.7)	13.5 (95% CI, 8.8-NR)	10.7 (2.1–25-7)
Kroiss M 2012 [4]	Phase II open label	Sunitinib	39	61	PFS	0	13	2.8	5.4 (95%CI, 3.2–7.6)	NA
Miller 2020 [12]	Prospective	Cabozantinib/Lenvatinib + Pembrolizumab	8	75	NA	25	63	6.3 (95%CI, 0.8-NR)	17.2 (95%CI, 3.6-NR)	83.0
Tingwei Su 2023 [7]	Retrospective	Anlotinib + Tislelizumab	37	NA	ORR	35	78.4	8.2 (95%CI, 5.5–15.2)	30.6 (95%CI, 21.1-NR)	NA
Kroiss M 2020 [5]	Retrospective	Cabozantinib	16	NA	PFS, OS	18	50	3.74 (0.65–14.09)	13.40 (1.29–19.19)	NA
Mert 2024 [9]	Retrospective	Cabozantinib/Lenvatinib + Pembrolizumab	17	NA	ORR, DCR, PFS, OS, safety	13	58.8	7.1 (0.7–66.4)	21.3 (3.3–70.3)	NA
Wendler J 2018 [8]	Retrospective	Cabozantinib	7	100	PFS	28	57	4.62 (0.69–12.47)	9.47 (3.00–18.71)	NA
Sara Bedrose 2020 [10]	Retrospective	Lenvatinib + Pembrolizumab	8	75	ORR	25	37.5	5.5 months (95%CI, 1.8-NR)	NA	NA

## Supplementary Materials

The following supporting information can be downloaded at: https://www.mdpi.com/article/10.3390/cancers17122004/s1, Table S1. PRISMA Checklist. Ref. [30] is cited in the Supplementary; Table S2. Search algorithm; Table S3. Quality appraisal of included studies according to MINORS assessment tool; Figure S1. Funnel Plot for ORR; Figure S2. ORR Sensitivity analysis; Figure S3. Bar plot illustrating efficacy outcomes by treatment in advanced ACC; Figure S4. Forest plot showing the proportion of patients surviving at 6 months across studies evaluating MKI monotherapy (MKI) and MKI plus immune-oncology (MKI + IO) in advanced ACC; Figure S5. Forest plot showing the proportion of patients surviving at 12 months across studies evaluating MKI and MKI + IO in advanced ACC; Figure S6. Forest plot showing the proportion of patients surviving at 6 months across studies evaluating MKI and MKI + IO in advanced ACC; Figure S7. Forest plot showing the proportion of patients surviving at 12 months across studies evaluating MKI and MKI + IO in advanced ACC.
