# Supplementary material for: The Efficacy and Safety of Multi-Kinase Inhibitors in Adrenocortical Carcinoma: A Systematic Review and Single-Arm Meta-Analysis
Source: Cancers (Basel). 2025 Jun 16;17(12):2004. doi: 10.3390/cancers17122004 (PMC12190831; doi:10.3390/cancers17122004)

Supplementary Table S1. PRISMA Checklist. From: Page MJ, McKenzie JE, Bossuyt PM, Boutron I, Hoffmann TC, Mulrow CD, et al. The PRISMA 2020 statement: an updated guideline for reporting systematic reviews. BMJ 2021;372:n71. doi: 10.1136/bmj.n71. This work is licensed under CC BY 4.0. To view a copy of this license, visit https://creativecommons.org/licenses/by/4.0/

Section and Topic	Item #	Checklist item	Location where item is reported
**TITLE**			
Title	1	Identify the report as a systematic review.	Title
**ABSTRACT**			
Abstract	2	See the PRISMA 2020 for Abstracts checklist.	Abstract
**INTRODUCTION**			
Rationale	3	Describe the rationale for the review in the context of existing knowledge.	1.Introduction
Objectives	4	Provide an explicit statement of the objective(s) or question(s) the review addresses.	1.Introduction
**METHODS**			
Eligibility criteria	5	Specify the inclusion and exclusion criteria for the review and how studies were grouped for the syntheses.	2.2 Inclusion Criteria
Information sources	6	Specify all databases, registers, websites, organisations, reference lists and other sources searched or consulted to identify studies. Specify the date when each source was last searched or consulted.	2.1 Registration and search study
Search strategy	7	Present the full search strategies for all databases, registers and websites, including any filters and limits used.	2.3 Data Extraction and definitions; Supplementary Table 1;
Selection process	8	Specify the methods used to decide whether a study met the inclusion criteria of the review, including how many reviewers screened each record and each report retrieved, whether they worked independently, and if applicable, details of automation tools used in the process.	2.4 Risk of bias assessment
Data collection process	9	Specify the methods used to collect data from reports, including how many reviewers collected data from each report, whether they worked independently, any processes for obtaining or confirming data from study investigators, and if applicable, details of automation tools used in the process.	2.1 Registration and search study
Data items	10a	List and define all outcomes for which data were sought. Specify whether all results that were compatible with each outcome domain in each study were sought (e.g. for all measures, time points, analyses), and if not, the methods used to decide which results to collect.	2.5 Statistical analysis
	10b	List and define all other variables for which data were sought (e.g. participant and intervention characteristics, funding sources). Describe any assumptions made about any missing or unclear information.	2.5 Statistical analysis
Study risk of bias assessment	11	Specify the methods used to assess risk of bias in the included studies, including details of the tool(s) used, how many reviewers assessed each study and whether they worked independently, and if applicable, details of automation tools used in the process.	2.4 Risk of bias assessment
Effect measures	12	Specify for each outcome the effect measure(s) (e.g. risk ratio, mean difference) used in the synthesis or presentation of results.	2.5 Statistical analysis
Synthesis methods	13a	Describe the processes used to decide which studies were eligible for each synthesis (e.g. tabulating the study intervention characteristics and comparing against the planned groups for each synthesis (item #5)).	2.5 Statistical analysis
	13b	Describe any methods required to prepare the data for presentation or synthesis, such as handling of missing summary statistics, or data conversions.	2.5 Statistical analysis
	13c	Describe any methods used to tabulate or visually display results of	2.5.1 – 2.5.8

Section and Topic	Item #	Checklist item	Location where item is reported
		individual studies and syntheses.	
	13d	Describe any methods used to synthesize results and provide a rationale for the choice(s). If meta-analysis was performed, describe the model(s), method(s) to identify the presence and extent of statistical heterogeneity, and software package(s) used.	2.5.1 – 2.5.8
	13e	Describe any methods used to explore possible causes of heterogeneity among study results (e.g. subgroup analysis, meta-regression).	2.5.1 – 2.5.8
	13f	Describe any sensitivity analyses conducted to assess robustness of the synthesized results.	2.5.1 – 2.5.8
Reporting bias assessment	14	Describe any methods used to assess risk of bias due to missing results in a synthesis (arising from reporting biases).	2.5.1 – 2.5.8
Certainty assessment	15	Describe any methods used to assess certainty (or confidence) in the body of evidence for an outcome.	2.5.1 – 2.5.8
**RESULTS**			
Study selection	16a	Describe the results of the search and selection process, from the number of records identified in the search to the number of studies included in the review, ideally using a flow diagram.	3.1 Study selection
	16b	Cite studies that might appear to meet the inclusion criteria, but which were excluded, and explain why they were excluded.	2.1 Registration and search study
Study characteristics	17	Cite each included study and present its characteristics.	Supplementary Table 2
Risk of bias in studies	18	Present assessments of risk of bias for each included study.	Supplementary Table 4.
Results of individual studies	19	For all outcomes, present, for each study: (a) summary statistics for each group (where appropriate) and (b) an effect estimate and its precision (e.g. confidence/credible interval), ideally using structured tables or plots.	3.2 Efficacy analysis and Forrest Plot
Results of syntheses	20a	For each synthesis, briefly summarise the characteristics and risk of bias among contributing studies.	Supplementary Table 3; Supplementary Table 4;
	20b	Present results of all statistical syntheses conducted. If meta-analysis was done, present for each the summary estimate and its precision (e.g. confidence/credible interval) and measures of statistical heterogeneity. If comparing groups, describe the direction of the effect.	3.2 – 3.5
	20c	Present results of all investigations of possible causes of heterogeneity among study results.	4. Discussion
	20d	Present results of all sensitivity analyses conducted to assess the robustness of the synthesized results.	3.2 Efficay analysis and forrest plot
Reporting biases	21	Present assessments of risk of bias due to missing results (arising from reporting biases) for each synthesis assessed.	2.4 Risk of bias assessment; Supplementary Table 4
Certainty of evidence	22	Present assessments of certainty (or confidence) in the body of evidence for each outcome assessed.	3.2 – 3.5
**DISCUSSION**			
Discussion	23a	Provide a general interpretation of the results in the context of other evidence.	4. Discussion
	23b	Discuss any limitations of the evidence included in the review.	4. Discussion

Section and Topic	Item #	Checklist item	Location where item is reported
	23c	Discuss any limitations of the review processes used.	4. Discussion
	23d	Discuss implications of the results for practice, policy, and future research.	4. Discussion
**OTHER INFORMATION**			
Registration and protocol	24a	Provide registration information for the review, including register name and registration number, or state that the review was not registered.	2.1 Registration and search study
	24b	Indicate where the review protocol can be accessed, or state that a protocol was not prepared.	2.1 Registration and search study;
	24c	Describe and explain any amendments to information provided at registration or in the protocol.	2.1 Registration and search study;<br>PROSPERO Protocol
Support	25	Describe sources of financial or non-financial support for the review, and the role of the funders or sponsors in the review.	Funding
Competing interests	26	Declare any competing interests of review authors.	Conflict of Interest
Availability of data, code and other materials	27	Report which of the following are publicly available and where they can be found: template data collection forms; data extracted from included studies; data used for all analyses; analytic code; any other materials used in the review.	The following materials<br>are publicly available as<br>Supplementary Files:<br>- Data extraction forms<br>used during the review<br>process (Supplementary<br>Table 2).<br>- Extracted data from<br>included studies<br>(Table 1 in the main text);<br>- Analytic code used for<br>Single-arm meta-analysis and<br>graphical outputs<br>(available at "available<br>upon request)<br>- PRISMA flowchart<br>(Supplementary Table 1

Section and Topic	Item #	Checklist item	Location where item is reported
			and Fig.1 in main text). All materials used in the review are available upon request from the corresponding author;

Supplementary Table S2. Search algorithm.

Pubmed
(adrenocortical OR adrenal) AND (cabozantinib OR lenvatinib OR apatinib OR sunitinib OR sorafenib OR axitinib OR imatinib OR dovitinib OR multikinase OR MKI OR TKI OR tyrosine kinase)

Supplementary Table S3 Quality appraisal of included studies according to MINORS assessment tool.

Study	A clearly stated aim	Inclusion of consecutive patients	Prospective collection of data	Endpoints appropriate to the aim of the study	Unbiased assessment of the study endpoint	Follow-up period appropriate to the aim of the study	Loss to follow up less than 5%	Prospective calculation of the study size	Overall Appraisal score
Yu-Chun Zhu 2024	2	2	2	2	1	2	2	2	15
O'Sullivan 2014	2	2	2	2	1	2	2	2	15
Campbell 2024	2	2	2	2	2	2	2	2	16
Enrique Grande 2024	2	2	2	2	1	2	2	2	15
Kroiss M 2012	2	2	2	2	1	2	2	2	15
Miller 2020	2	2	2	2	1	2	2	2	15
Tingwei Su 2023	2	1	0	2	2	2	2	0	11
Kroiss M 2020	2	1	0	2	1	2	2	0	10
Mert 2024	2	1	0	2	1	2	2	0	10
Wendler J 2018	2	1	0	2	1	2	2	0	10
Sara Bedrose 2020	2	1	0	2	1	2	2	0	10

Supplementary Figure S1. Funnel Plot for ORR. Funnel and Egger's test (p=0.0083) indicated potential publication bias, with a significant asymmetry likely reflecting a bias toward positive findings in this rare disease setting. Begger test: Linear regression test of funnel plot asymmetry. Test result: t=-3.48, df=8, p-value=0.0083. Bias estimate: -3.0792 (SE=0.8848). Multiplicative residual heterogeneity variance (tau^2 = 1.0537).

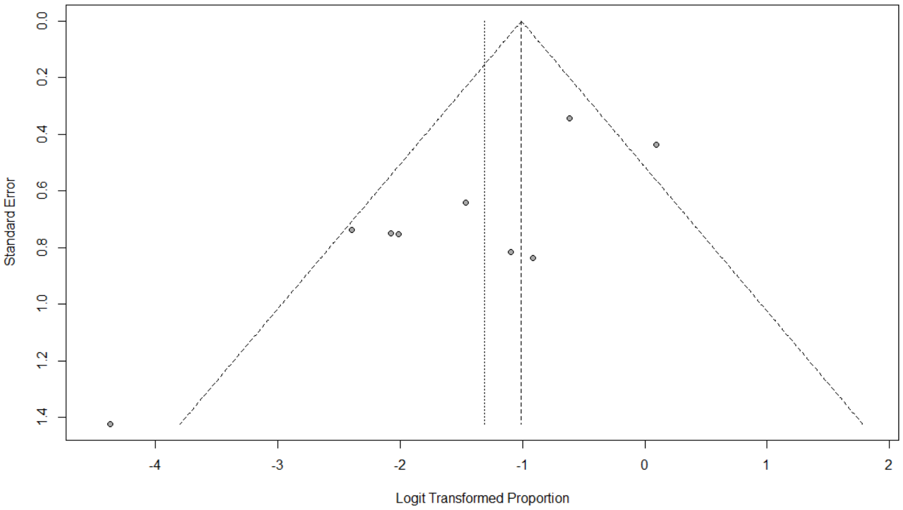

Supplementary Figure S2. ORR Sensitivity analysis. Forest plot showing pooled ORR across included studies evaluating MKI-based therapies in advanced ACC. The random-effects model demonstrated substantial heterogeneity (I² = 77.2%; p = 0.0015), with an overall pooled ORR of 16% (95% CI: 5%-41%). Each study's proportion of responders, total number of patients (N), and respective 95% confidence intervals (CIs) are represented individually.

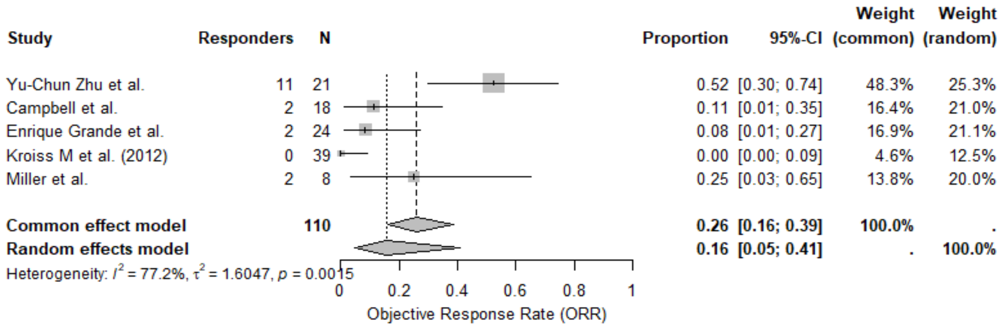

Supplementary Figure S3. Bar plot illustrating efficacy outcomes by treatment in advanced ACC. Objective Response Rate (ORR, %), Overall Survival (OS, months), and Progression-Free Survival (PFS, months) are shown for each regimen. Combinations of MKIs with immune checkpoint inhibitors, particularly apatinib plus camrelizumab, demonstrated the highest ORR and prolonged PFS, while cabozantinib monotherapy was associated with the longest OS.

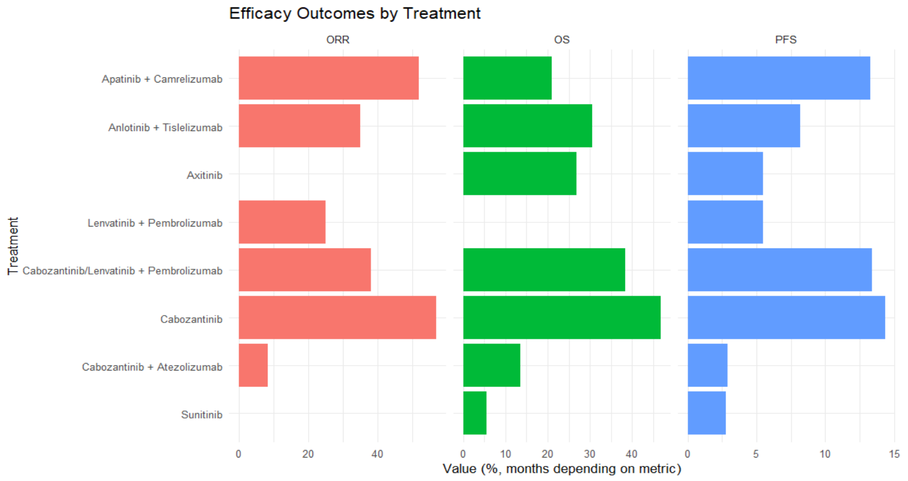

Supplementary Figure S4. Forest plot showing the proportion of patients surviving at 6 months across studies evaluating MKI monotherapy (MKI) and MKI plus immune-oncology (MKI+IO) in advanced ACC. The pooled 6-month survival was higher in the MKI+IO group (65%, 95% CI: 53-76%) compared to the MKI group (47%, 95% CI: 36–57%). A significant difference between subgroups was observed (p = 0.0213). Random-effects models were used to account for inter-study variability.

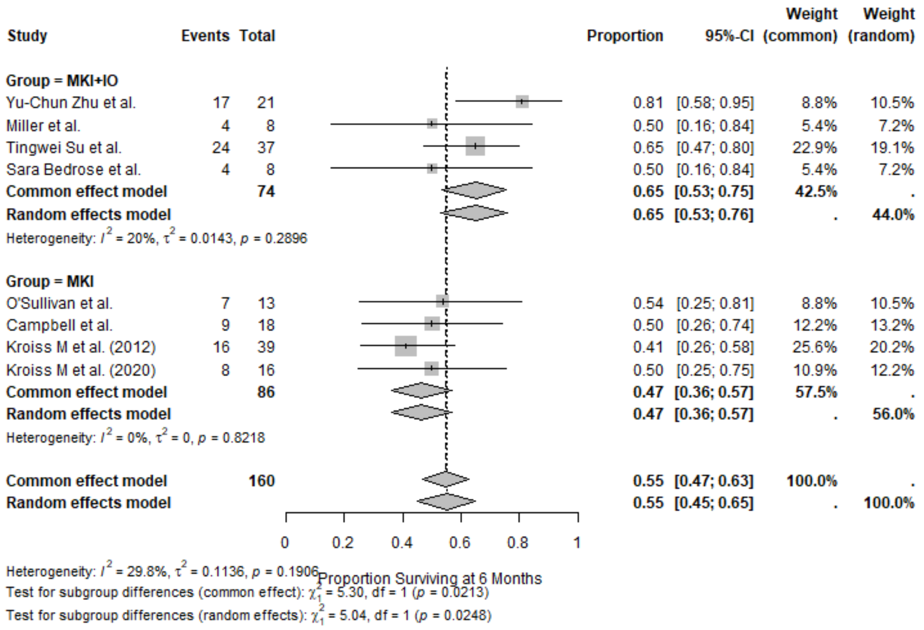

Supplementary Figure S5. Forest plot showing the proportion of patients surviving at 12 months across studies evaluating MKI and MKI+IO in advanced ACC. The pooled 12-month survival was 44% (95% CI, 33-55%) in the MKI+IO group and 30% (95%CI, 22-41%) in the MKI group. Although a trend toward improved survival with MKI+IO was observed, the difference between subgroups was not statistically significant (p=0.0890). Random-effects models were used to account for inter-study variability.

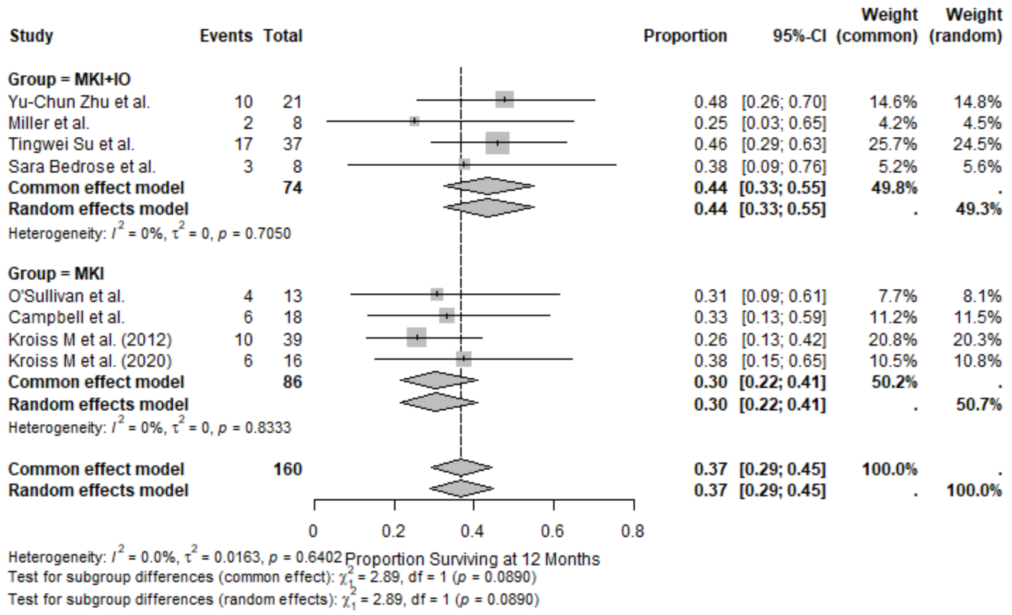

Supplementary Figure S6. Forest plot showing the proportion of patients surviving at 6 months across studies evaluating MKI and MKI+IO in advanced ACC. The pooled 6-month survival was 84% (95%CI, 73-91%) for the MKI+IO group and 76% (95%CI, 65-85%) for the MKI group. No statistically significant difference was observed between subgroups (p=0.2148). A random-effects model was used, with no significant heterogeneity detected across studies.

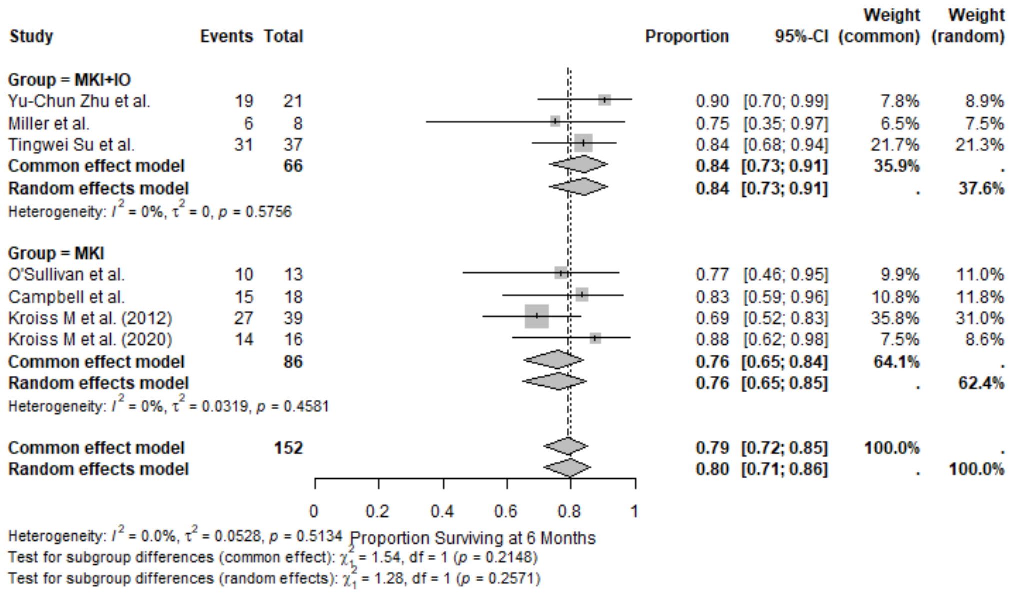

Supplementary Figure S7. Forest plot showing the proportion of patients surviving at 12 months across studies evaluating MKI and MKI+IO in advanced ACC. The pooled 12-month survival was 67% (95%CI, 54-77%) in the MKI+IO group and 58% (95%CI, 46-70%) in the MKI group. No statistically significant difference between subgroups was observed (p=0.2730). Random-effects models were applied, with minimal heterogeneity across studies.

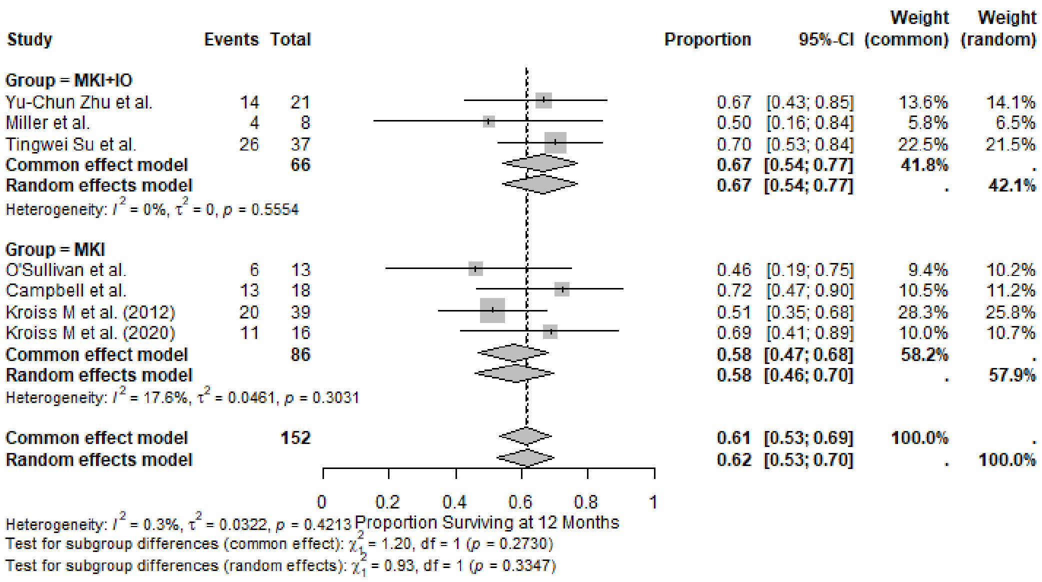

Supplement: Supplementary file 1 [file cancers-17-02004-s001.zip › cancers-3665072-supplementary.pdf]
